# Editorial: Computational methods for protein characterization: in memoriam of Donald Abraham

**DOI:** 10.3389/fmolb.2023.1289441

**Published:** 2023-09-15

**Authors:** Francesca Spyrakis, Glen E. Kellogg

**Affiliations:** ^1^ Department of Drug Science and Technology, University of Turin, Turin, Italy; ^2^ Department of Medicinal Chemistry and Drug Discovery, Institute for Structural Biology, Drug Discovery and Development, Virginia Commonwealth University, Richmond, VA, United States

**Keywords:** Donald Abraham, haemoglobin, structural biology, HINT, protein modelling

This Research Topic of Frontiers in Molecular Biosciences is dedicated to the loving memory of Professor Donald J. Abraham. After a successful career at the University of Pittsburgh, Donald was recruited to be the Chair of the Department of Medicinal Chemistry (1988–2000) at the Virginia Commonwealth University, where his passion and enthusiasm led to him being the Founding Director of the Institute for Structural Biology and Drug Discovery at VCU, a position he held between 1995 and 2007. During his long career he worked with Professor Alfred Burger (as a postdoctoral at the University of Virginia) and notably with Professor Max Perutz at the Medical Research Council laboratory of the University of Cambridge. This collaboration led to the development of hemoglobin (Hb) allosteric effectors such as drugs to treat sickle cell disease (SCD) and other disorders. He was the editor of the two most recent editions of Burger’s Medicinal Chemistry, and, in 2010, he was inducted into the Medicinal Chemistry Hall of Fame, a distinguished honor bestowed by the American Chemical Society’s Division of Medicinal Chemistry. He also received the Honorary Degree in Medicinal Chemistry from the University of Parma.

Since very early in his career, his vision was that structural biology and computer-aided approaches were fundamental for drug discovery and design, and he strongly supported the development of these methodologies and of scientists working with him to become international experts in the field. Glen Kellogg joined Abraham at VCU in 1988 as a postdoctoral and began the collaboration leading to HINT, the first force field for evaluating molecular interaction energetics, simply and intuitively based on LogP. During the following years, HINT applications helped us to better understand the nature of protein-protein, protein-DNA and protein-ligand interactions, as well as the role of pH and waters in mediating molecular recognition. Since that start, HINT has generated more than 100 articles, and garnered thousands of citations.

The group of the University of Parma had the fortune of contributing to the development of HINT via visiting, projects and many publications.

This Research Topic, entitled *Computational Methods for Protein Characterization: In Memoriam of Donald Abraham*, contain two original research work and three reviews, by scientists who have worked with him and others who have indirectly known him because of his outstanding and last contributions to research.

• Babbi et al. demonstrate that physicochemical grouping of residue variations in proteins can help in determining whether patterns of variation types are related to specific groups of diseases, thus helping to associate gene variants with genetic diseases.
• AL Mughram et al. recorded the interaction of hydrophobic residues in a large set of soluble and membrane proteins as three-dimensional maps. The latter, once clustered, compose a library of interaction profiles encoding interaction strengths, interaction types and the optimal 3D position for interacting residue-residue partners. The end game is exploiting these data in structure prediction and modeling.
• In a following contribution, Donkor et al., review structural biology, X-ray crystallography and structure-based drug discovery from the perspective of Hb. They present the impact of X-ray crystallography in SCD drug development using Hb as a target, emphasizing the major and important contributions by Don Abraham in this field.
• Kellogg et al., who all spent most of our careers collaborating with Donald, proudly describe and summarize the results of the extensive and successful collaboration between Donald and Glen at VCU and the group at the University of Parma for testing HINT in a variety of different biomolecular interactions, from proteins with ligands to proteins with DNA.
• Lastly, Purisima et al. report about the Solvated Interaction Energy (SIE) scoring function, a physics-based method that arose from efforts to understand the physics governing binding events, with particular care given to the role played by solvation. They review successful applications of SIE in virtual screening and discovery of novel small-molecule binders, as well as in the optimization of known drugs.

Donald Abraham was an inspiration for almost everyone who had an opportunity to work with him. Because he had more ideas before breakfast than the rest of us would have in a week, there was always a buzz and enthusiasm when he was around. He will be missed, but his contributions to medicinal chemistry and structural biology, and especially their intersection, are permanent.

